# Mental Health of Teachers Who Have Teleworked Due to COVID-19

**DOI:** 10.3390/ejihpe11020037

**Published:** 2021-06-09

**Authors:** Claudia Palma-Vasquez, Diego Carrasco, Julio C. Hernando-Rodriguez

**Affiliations:** 1 Faculty of Education, Universidad Católica de la Santísima Concepción, Concepción 4070129, Chile; 2 Center for Research in Occupational Health (CiSAL), Department of Experimental and Health Sciences, Pompeu Fabra University, 08003 Barcelona, Spain; julio.hernando@upf.edu; 3 Centro de Medición MIDE UC, Pontificia Universidad Católica de Chile, Santiago 7820436, Chile; dacarras@uc.cl

**Keywords:** mental health, COVID-19, teachers, forced telework, GHQ-12, Rasch model

## Abstract

The impact of the COVID-19 pandemic on education included school closures and the implementation of virtual teaching and teleworking without the knowledge or resources needed to do so. This situation accentuated the inequality in accessing quality education and generated high rates of stress, anxiety, and general discomfort in teachers. This study aimed to explore the mental health of teachers who were forced to telework because of COVID-19, and to analyze the association with sociodemographic, teacher-related, and working conditions. The sample was 278 classroom teachers in Chile who teleworked more than 50% during the 2020 academic year. The participants were mostly women (82%) who entered the teaching profession at age 30 or younger (87%) and worked two or more unpaid overtime hours per day (67%). The dependent variable was mental health measured through the General Health Questionnaire (GHQ-12). The independent variables were sociodemographic, teacher-related, and work conditions. The internal structure of the mental health construct was evaluated using the Rasch model. Crude odds ratios (cORs) and adjusted odds ratios (aORs) were estimated using logistic regression models. A high rate of poor mental health was identified in teachers (58%). The variables associated with poor mental health were working in a private–subsidized school (aOR = 2.89; 95% CI: 1.16–7.22), working two or more unpaid overtime hours (aOR = 2.25; 95% CI: 1.11–4.59), and being absent due to sickness (aOR = 3.82; 95% CI: 1.53–9.58). These results provide evidence suggesting the need for actions to improve the working conditions of teachers who telework in order to improve their mental health, and thus have a positive impact on the entire educational community.

## 1. Introduction

The pandemic caused by COVID-19 has impacted all societies and has presented an unprecedented challenge to global health, the environment, the economy, and education. From the end of March 2020, many countries around the world were confined, with the aim of stopping the spread of the disease. Telework from home, defined by the International Labour Organization (ILO) as “work done at home using electronic equipment” [1], has been the mechanism through which multiple economic activities have continued to develop. The educational field is one of the most affected social sectors since the pandemic forced the suspension of face-to-face teaching activities and led to virtual or distance learning and teleworking. The situation in Latin America and the Caribbean is especially worrying since the duration of school closures has been longer than in any other region in the world [2]. In Chile, the suspension of activities took place at the beginning of the academic year (i.e., from March 2020), which meant that face-to-face teaching was suspended for practically all of the 2020 academic year. The suspension of face-to-face activities caused an average loss of 88% of annual learning for students, which increased up to 95% for the most disadvantaged students [3]. For teachers, the situation is equally complex, even though they are among the groups that mostly kept their jobs due to teleworking; for many of them it meant facing multiple challenges without the knowledge or resources needed to do so. Nevertheless, the regulation of telework in March 2020 [4] may have contributed to improving the working conditions of some teachers in Chile, since it stipulates that the employer is responsible for providing work equipment, tools, or materials. It must be considered that this law modifies the labor code that regulates the recruitment and working conditions of private school teachers, while public school teachers are subjected to the Teacher’s Statute [5], which does not currently provide for the regulation of telework. 

The Chilean educational system is an interesting scenario for research, because in Chile, as in various other countries, there is a mix of schools with public, private, and mixed financing (i.e., private–subsidized) [6]. Public schools are fully funded by the government and are open to all students at no cost. Private schools are privately financed and can be accessed by those who can pay for them. Mixed funding refers to private schools that are subsidized by the government according to the number of students. Previous studies have shown that large differences in student achievement may be related to differences in their socioeconomic background and type of school they attend [7].

Teachers in Chile are a group of workers who have high turnover and dropout rates, as in some parts of the world [8], even reaching 20% in the first year [9]. The literature indicates that some of the reasons for high teacher mobility are low salaries, poor working conditions [10,11], the vulnerability of students, temporary contracts, and fewer contract hours that translate into lower income [12]. Leaving the teaching profession early has a negative and unequal impact on the educational system, affecting the most disadvantaged areas. It seems reasonable to suppose that unfavorable working conditions due to the improvised use of teleworking during the pandemic, with the consequent use of one’s own resources, difficulty in communicating with students, and a lack of coordination between teachers and school administrators, may have exacerbated the job dissatisfaction that already existed among teachers before the pandemic. 

This complex scenario, as a result of changes in the usual way of teaching along with the amplified economic, structural, and competence heterogeneity of schools and teachers due to the pandemic, has accentuated the existing inequality in access to quality education [13] and generated high levels of stress, anxiety, and general discomfort among teachers [14,15]. The aforementioned studies showed that a lack of connection and sufficient digital skills in online teaching were related to stress and poor mental health, while support from coworkers and having better coping strategies were protective factors. In this sense, recent studies indicate that the instability, uncertainty, and fear caused by the COVID-19 pandemic have alarmingly increased poor mental health [16]. Poor mental health is a widespread problem that affects not only the people who suffer with it, but also their families, their work environment, and the educational system. As mentioned above, the implementation of forced telework without adequate work conditions may have had an influence on the mental health or well-being of teachers, which, in turn, may have impacted the quality of education they provided to their students [17,18]. We identified a gap in the literature on the mental health status of teachers who work remotely in the context of a pandemic. For these reasons, we believe that it is essential to know the status of teachers’ mental health, paying special attention to experience and gender, the former because less experience is a factor that seems to have an impact on higher turnover [8], and the latter because women may have an additional workload in terms of domestic or children care tasks according to stereotyped gender roles [19] that could be magnified during a pandemic.

We posed two research questions to address the aim of this study: How many teachers have worse mental health? What are their main characteristics? We hypothesized that there could be a high prevalence of poor mental health among teachers in Chile and that the worst sociodemographic, teacher-related, and working conditions could be related to this. In particular, we supposed that working in a public school, having less teaching experience, being a “head teacher” (because of the additional workload of having responsibility for tutoring students apart from teaching in one’s discipline) [20], temporary contracts, long working hours, unpaid overtime hours, and absence due to sickness could be related to worse mental health. Likewise, we supposed that having less teaching experience and being a woman could be related to a higher risk of poor mental health. The aim of the study was to describe mental health and analyze its relationship with sociodemographic, teacher-related, and working conditions among a sample of teachers from two regions in Chile (Bío-Bío and Ñuble) who were forced to telework in inadequate conditions during the 2020 academic year due to the COVID-19 pandemic.

## 2. Materials and Methods

The study design is predictive cross-sectional [21].

### 2.1. Procedure

A total of 711 teachers from two regions of Chile (Ñuble and Bío Bío) who had already answered the Encuesta de Trayectoria Laboral Docente (E-TLD) in 2018 and/or 2019 were invited to participate to complete the 2020 version. The data collection was conducted online between December 2020 and January 2021, and the questions contained in the survey referred to the 2020 academic year.

### 2.2. Participants

Teachers who (i) worked as classroom teachers, (ii) reported teleworking more than 50% during the 2020 academic year, and (iii) gave informed consent to participate were included in the study, resulting in 278 participants in total. The sociodemographic characteristics show that the sample group was mainly composed of women (81.65%), they entered the teaching profession when they were 30 years old or younger (86.56%), they were in the Bío Bío region (76.26%), and their educational institutions were mainly private–subsidized (47.48%) (Table 1). The descriptions of the variables related to the teachers indicate that they worked mainly in primary education (52.88%), were head teachers (69.78%), and had more than 10 years of teaching experience (43.80%). The most frequent working conditions were permanent contract (71.58%), working more than 35 hours per week (79.14%), working 2 or more unpaid overtime hours per day due to online teaching because of the pandemic (66.55%), and not being absent due to sickness during the 2020 academic year (83.81%).

### 2.3. Ethical Considerations

This study was part of the doctoral project of the principal author, which was approved by the Ethics Committee of the Universidad Católica de la Santísima Concepción, Chile. Participation in this study was completely voluntary, and participants were informed and guaranteed confidentiality for the use and treatment of the data. The fundamental ethical principles of the Belmont Report [22] and the Declaration of Helsinki [23] were considered.

### 2.4. Variables

The dependent variable of this study was mental health, measured through Goldberg’s General Health Questionnaire (GHQ-12) [24]. The GHQ-12 is one of the most widely used self-administered instruments to detect the risk of developing psychiatric disorders or health problems such as depression or anxiety. This instrument has 12 questions scored on a Likert scale from 1 to 4 regarding psychological discomfort (items 1–6: 1 = never, 4 = always) and psychological well-being (items 7–12: 1 = always, 4 = never). The responses to the items were coded using the standard method (0011), which dichotomizes the scoring as 0 (scores of 1 and 2) and 1 (scores of 3 and 4). These responses were summed up to obtain a total score, with values ranging from 0 to 12. A higher score on the scale indicates the presence of poor mental health. A total score ≥3 indicates a greater risk of poor mental health [25,26,27].

The independent variables were sociodemographic variables: gender (male, female), age at entry into the teaching profession (≤30, 31–40, ≥41), region (Bío-Bío, Ñuble, other), and type of school (private, private–subsidized, public). The variables related to teachers were the educational teaching level (preschool, primary, secondary), being a head teacher (no, yes), and years of teaching experience (≤5, 6–10, >10). Working conditions included the type of contract (permanent, fixed-term for more than 1 school year, fixed-term for 1 school year or less), weekly working hours (≤35, >35), unpaid overtime hours (none, <2 hours per day, ≥2 hours per day) and being absent due to sickness during 2020 (no, yes).

### 2.5. Statistical Analysis

First, we conducted a descriptive analysis (means, standard deviations, and frequencies of response categories) of the items of the mental health scale (GHQ-12). Second, we dichotomized the GHQ-12 scores. Third, we calculated the internal consistency index of the corrected scoring scale through Cronbach’s alpha. Fourth, we evaluated the internal structure and content validity of the mental health scale [28]. For that purpose, we fitted a Rasch model based on item response theory (IRT) [29], then we contrasted this model with the expected distribution of the participants’ responses using a person-item map [30]. The distribution of items along the scale guided the interpretation of the scores generated, constituting a validity argument regarding the instrument used [31]. Previous studies indicate that there is no adequate absolute fit index, but indices between 0.75 and 1.33 are considered acceptable [32]. Fifth, we calculated the percentage of participants with a score ≥3 on the GHQ-12 scale to identify the proportion of teachers with worse mental health. Finally, we performed logistic regression analyses to obtain crude odds ratios (cORs) and adjusted odds ratios (aORs) with a 95% confidence interval to identify the extent to which sociodemographic variables related to teachers and working conditions can predict poor mental health. Likewise, we stratified the analyses according to groups by years of experience and gender to identify vulnerable groups. Rasch analysis was performed with Mplus version 8.3 and R software, and descriptive, internal consistency, and logistic regression analyses were performed with Stata v.13.

## 3. Results

The frequencies, mean scores, and standard deviations for the responses to the items of the GHQ-12 mental health scale are described in Table 2. These results show that there was a low perception of having no value as a person (item 6: mean 1.64, SD 0.80) and a high perception of being under strain (item 2: mean 2.73, SD 0.85). 

The prevalence of poor mental health among teachers was 58.27% and the sum score of the 12 questions had a mean of 3.76 points with a standard deviation of 3 (Table 3).

**Figure 1:**
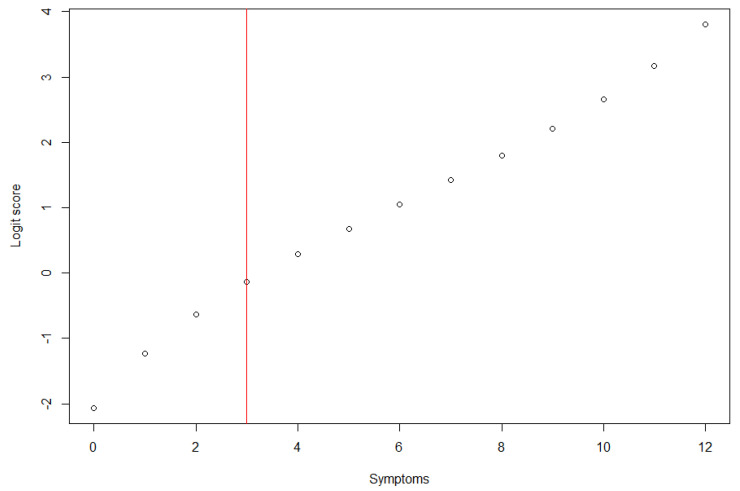
Score (symptoms) of dichotomized mental health scale (GHQ-12) corresponding to logit score.

The analysis of internal consistency through Cronbach’s alpha indicated high reliability of the scale (α = 0.81). The results of the Rasch IRT model showed an adequate fit of the data, which allowed an interpretation of mental health through the proposed single score measure (INFIT between 0.88 and 1.20) (Figure 1).

**Figure 2:**
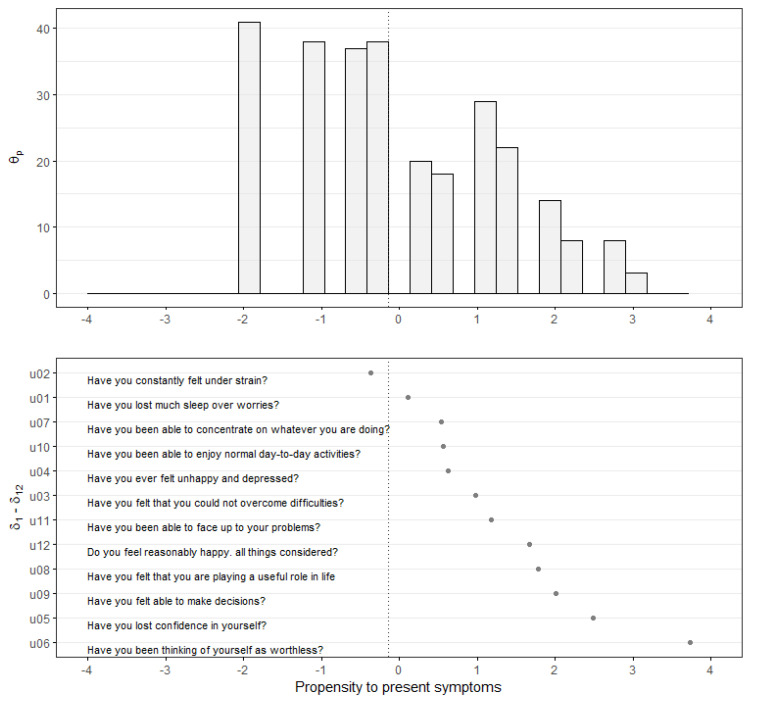
Person-item map (propensity to present symptoms) of dichotomized scale of poor mental health (GHQ-12) in logits.

The person-item map allows us to show the propensity with which teachers present different mental health symptoms, which ones are more frequent and which are more severe. Regarding the symptoms reported by those whose score exceeded the cut-off of ≥3, more than half the teachers indicated that they constantly felt under strain. In addition to those mentioned above, the three most frequent symptoms were losing sleep because of worry, having difficulty concentrating, and having difficulty enjoying normal day-to-day activities. In contrast, the three most severe symptoms were thinking of oneself as worthless, losing confidence in oneself, and losing decision-making ability (Figure 2).

We evaluated whether the missing data were distributed completely at random (MCAR) for the variable “age at entry into the teaching profession” (7.6%) by applying Little’s MCAR test [33], and the result showed that they were not (*p* < 0.05). Applying additional analysis, a missing variable in age was created and the χ2 test was performed with all variables. This showed that the missing data had no significant relationship with the dependent variable of mental health (*p* = 0.81), or with the majority (8 out of 10) of the independent variables [34]. Therefore, it could be assumed that the missing data were randomly distributed (MAR) and relied on subsequent logistic estimations [35].

Logistic regression analysis was applied for all participants without missing data for any of the variables considered. The total sample was made up of 251 participants. The results of logistic regression analysis show that teachers who worked in private–subsidized schools were 189% more likely to have poor mental health compared to those who worked in private schools (aOR = 2.89; 95% CI: 1.16–7.22). The risk of having poorer mental health was higher for those who worked two or more unpaid overtime hours per day compared to those who did not need to extend their working hours during the pandemic (aOR = 2.25; 95% CI: 1.11–4.59). Being absent due to sickness was associated with a 282% greater likelihood of having poor mental health compared with not being absent during 2020 (aOR = 3.82; 95% CI: 1.53–9.58) (Table 4).

The results of stratified analysis by years of experience (n = 242) show that, in the group with 6–10 years of teaching experience, entering the teaching profession at age 30 or younger (aOR = 9.77; 95% CI: 1.17–81.21), working two or more unpaid overtime hours (aOR = 6.11; 95% CI: 1.09–34.30), and being absent due to sickness during 2020 (aOR = 6.72; 95% CI: 1.04—43.61) were associated with poorer mental health. However, working more than 35 hours per week was associated with a lower risk of poor mental health (aOR = 0.07; 95% CI: 0.01–0.51). Among teachers with 5 years or less of work experience, having a fixed-term contract for one school year or less was positively associated with poorer mental health compared to having a permanent contract (aOR = 90.05; 95% CI: 1.25–6473.93) (Table 5).

Finally, the results stratified by gender are only shown for women (n = 210), as the sample of men was too small to be interpretable (Table 6). Among women, being a head teacher reduced the risk of poor health compared to being a classroom teacher (aOR = 0.48; 95% CI: 0.23–1.0). The female teachers who worked two or more unpaid overtime hours per day (aOR = 4.40; 95% CI: 1.86–10.39) and those who were absent due to sickness during 2020 (aOR = 3.08; 95% CI: 1.19–7.97) were at higher risk of having poor mental health.

## 4. Discussion

This study shows a high prevalence of poor mental health in this sample of teachers in Chile who were forced to telework without the knowledge or resources needed to do so during the COVID-19 pandemic. In general, working in private–subsidized schools, working two or more unpaid overtime hours per day, and being absent due to sickness during 2020 were associated with poorer mental health. In the group with 6 to 10 years of teaching experience, the results were similar, although of greater magnitude. In the group with 5 years or less of teaching experience, the shorter contract duration was a predictor of poor mental health. Working more than 35 hours per week (in the group with 6 to 10 years of experience) and being a head teacher (in the group of women) were protective factors of mental health.

In the non-pandemic context, contrary to what might be thought, teachers do not experience poorer mental health compared to other professions [36]. Unfortunately, the absence of comparative studies on the effects of telework in different professions during a pandemic makes it impossible to identify whether teachers experience worse mental health compared to other workers. However, according to recent studies, the pandemic appears to have increased the level of poor mental health among teachers [15,37]. Our finding is consistent with such studies and with research that show that in a pandemic, teachers perceive more stressors (e.g., worry about their professional future), have poorer well-being [38], and experience more feelings of grief, distress, and stress [39].

We identified that some sociodemographic and teacher characteristics, as well as some working conditions, act as predictors of poor mental health. In this sense, the type of school is a predictor of poor mental health. In particular, working in a private–subsidized school was related to having poorer mental health, which may be because in Chile these types of schools tend to have a concentration of worse educational conditions [40], worse salary conditions due to a less regulated professional career [41], and higher rates of teacher turnover [42]. These findings may be useful to understand what happens in other countries where there are different types of schools such as public, private, and mixed financing. That those teachers who work two or more unpaid overtime hours per day have poorer mental health was an expected result. According to previous studies, there is an association between long working hours and health problems [43], increased stress, and poorer psychological well-being [44]. Furthermore, an Argentinean study in the context of the pandemic showed that teleworking increases the daily working hours and accentuates material inequalities among teachers [45]. Being absent due to sickness also turns out to be a predictor of poor mental health, because people usually miss work when their health condition makes it impossible for them to be there, which is consistent with previous studies in the general working population [26,46].

In the group of teachers with less than 5 years of work experience, working conditions, such as shorter contract duration, was a predictor of poorer mental health. However, the estimate was not very accurate, as it was within a wide confidence interval. In contrast, in the group of teachers with 6 to 10 years of experience, longer working hours was a protective factor for mental health. Previous studies indicate that temporary or short-term contracts [47] and long working hours [48] have a negative impact on the health of working people. Moreover, studies of Chilean teachers who were beginning their professional careers indicate that, among other factors, working conditions such as an excessive workload that may force them to work overtime resulted in high rates of early-career dropout [10]. However, regarding the inverse relationship we found between long working hours and mental health, it is possible that personal satisfaction and the sense of identity that an educational career provides leads to greater resilience in a stressful work context [49]. Another explanation would be that the higher salary that a full working day provides allows the necessary economic stability to protect from worse mental health.

It is necessary to add that there is inequality with regard to mental health according to gender. Possibly women’s mental health is influenced more by the dual activities of work and home than by the working conditions, as is the case for men [19,50]. Nevertheless, contrary to what previous studies indicate, among women, being a head teacher was shown to be a protective factor for mental health. Before the pandemic in Chile, being a head teacher was related to work overload, stress, and emotional exhaustion [20]. However, during the pandemic, the more fluid online communication that head teachers have with students, parents, and families may facilitate an effective educational process. This could be a factor that reduces discomfort in the virtual education context [51].

As a limitation of the study, we cannot establish causality due to the cross-sectional design, although the data come from a longitudinal design. This is because the mental health scale was incorporated in the last wave of the survey. Regarding data collection, we identified as a limitation the lack of background information about family composition, since studies in the pandemic context have identified that knowledge of marital status and having children or not, among other characteristics, were relevant to explain the stress associated with telework for teachers [39]. Finally, the limited sample size for some stratified analyses resulted in high confidence intervals, which made interpretation difficult. As a strength, this is the first study to explore mental health in a sample of the teaching population in Chile with recent data derived from forced telework during 2020 due to the COVID-19 pandemic, using the GHQ-12 instrument and providing evidence of its validity for correct interpretation. Likewise, we can indicate that this study contributes significantly to the validity of studies using the mental health scale for the adequate interpretation of results among teachers in Chile. We provide evidence for a unidimensional interpretation of the mental health scale in this study, which is fundamental in health research [52].

This study has important theoretical and practical implications. Among the former, first, it provides evidence of validity for the interpretation of the GHQ-12 instrument used to measure mental health in teachers in Chile. Second, it reveals the state of mental health of teachers in the context of a pandemic, and finally, it indicates reasons that can be associated with worse mental health. Among the practical implications, this study could be useful when making educational policy decisions that can improve the working conditions of teachers, since they seem to be related to mental health. This could be of interest in other countries where the educational system is similar to Chile’s.

## 5. Conclusions

This study contributes to the recognition of a high prevalence of perceived ill health in teachers who have been forced to telework due to the COVID-19 pandemic. Our findings highlight that sociodemographic, teacher, and work contexts should be taken into consideration in order to study their relationship with teachers’ mental health. Although we advise caution since we cannot establish causality, these results suggest paying more attention to regularizing the workday so that there is a greater allocation of hours for teachers to plan their work. This could benefit their mental health, especially considering that virtual teaching is likely to be institutionalized or extended beyond the pandemic situation. Similarly, this study may be useful as a starting point for the development of support networks for teachers’ emotional well-being, which could also improve the mental health of the entire educational community.

## Figures and Tables

**Table 1:** Descriptive characteristics of sample (n = 278), Chile, 2020 (E-TLD).

Characteristics	Categories	n	%
Gender	Male	51	18.35
	Female	227	81.65
Age at entry into profession ^1^	>40	2	0.79
	31–40	32	12.65
	≤30	219	86.56
Region	Bío-Bío	212	76.26
	Ñuble	45	16.19
	Other	21	7.55
Type of school	Private	33	11.87
	Private–subsidized	132	47.48
	Public	113	40.65
Educational teaching level	Secondary	92	33.09
	Primary	147	52.88
	Preschool	39	14.03
Head teacher	No	84	30.22
	Yes	194	69.78
Years of teaching experience	>10	120	43.80
	6–10	92	33.58
	≤5	62	22.63
Type of contract	Permanent	199	71.58
	Fixed-term for >1 year	36	12.95
	Fixed-term for ≤1 year	43	15.47
Weekly working hours	≤35	58	20.86
	>35	220	79.14
Unpaid overtime hours	None	57	20.50
	<2 h daily	36	12.95
	≥2 h daily	185	66.55
Sickness absence during 2020	No	233	83.81
	Yes	45	16.19

^1^ 25 missing data items in the variable.

**Table 2:** Descriptive statistics of items of General Health Questionnaire (GHQ-12) (n = 278). Chile, 2020 (E-TLD).

Items	Mean	SD	Frequency of Response Categories (%)			
			1	2	3	4
Psychological discomfort						
1. Have you lost much sleep over worries? (u01)	2.55	0.88	9.71	42.09	32.01	16.19
2. Have you constantly felt under strain? (u02)	2.73	0.85	5.04	38.49	35.25	21.22
3. Have you felt that you could not overcome difficulties? (u03)	2.17	0.91	25.18	41.01	25.18	8.63
4. Have you ever felt unhappy and depressed? (u04)	2.31	0.88	18.35	42.09	29.86	9.71
5. Have you lost confidence in yourself? (u05)	2.31	0.88	53.60	32.01	11.51	2.88
6. Have you been thinking of yourself as worthless? (u06)	1.64	0.80	79.14	15.11	3.60	2.16
Psychological well-being						
7. Have you been able to concentrate on whatever you are doing? (u07)	2.26	0.83	20.14	38.85	36.33	4.68
8. Have you felt that you are playing a useful role in life? (u08)	1.77	0.88	48.92	28.78	18.71	3.60
9. Have you felt able to make decisions? (u09)	1.74	0.80	47.12	33.45	17.99	1.44
10. Have you been able to enjoy normal day-to-day activities? (u10)	2.19	0.87	25.90	33.45	36.33	4.32
11. Have you been able to face up to your problems? (u11)	1.99	0.86	34.17	35.25	27.70	2.88
12. Do you feel reasonably happy, all things considered? (u12)	1.87	0.80	38.13	38.13	22.30	1.44

**Table 3:** Mental health scale score (GHQ-12, dichotomous scoring) frequency (n = 278). Chile, 2020 (E-TLD).

Score	n	%	Accumulated %
0	41	14.75	14.75
1	38	13.67	28.42
2	37	13.31	41.73
3	38	13.67	55.40
4	20	7.19	62.59
5	18	6.47	69.06
6	29	10.43	79.50
7	22	7.91	87.41
8	14	5.04	92.45
9	8	2.88	95.32
10	8	2.88	98.20
11	3	1.08	99.28
12	2	0.72	100.00
Total	278	100.00	
Poor mental health (cut point ≥ 3)	n	%	
No	116	41.73	
Yes	162	58.27	

**Table 4:** Association between poor mental health and sociodemographic teacher-related variables and working conditions in Chilean teachers (n = 251). Chile, 2020 (E-TLD).

Characteristics	Categories	Total Sample			
		cOR	CI (95%)	aOR	CI (95%)
Gender	Male	1		1	
	Female	1.07	0.58–1.98	0.92	0.42–2.01
Age at entry into profession	>40	1		1	
	31–40	0.57	0.27–1.20	0.52	0.23–1.19
	≤30	-	-	-	-
Region	Bío-Bío	1		1	
	Ñuble	0.95	0.50–1.83	1.02	0.47–2.22
	Another	0.77	0.31–1.88	0.81	0.23–2.81
Type of school	Private	1		1	
	Private-subsidized	**2.78**	**1.27–6.09**	**2.89**	**1.16–7.22**
	Public	2.01	0.91–4.43	2.35	0.86–6.39
Educational teaching level	Secondary	1		1	
	Primary	0.92	0.54–1.57	0.89	0.47–1.69
	Pre-school	0.87	0.41–1.86	0.75	0.30–1.87
Head teacher	No	1		1	
	Yes	0.7	0.41–1.18	0.59	0.31–1.13
Years of teaching experience	>10	1		1	
	6–10	**1.9**	**1.09–3.34**	1.83	0.95–3.53
	≤5	1.53	0.82–2.86	2.26	0.96–5.34
Type of contract	Permanent	1		1	
	Fixed-term for >1 year	0.97	0.48–1.98	0.55	0.21–1.44
	Fixed-term for ≤1 year	1.79	0.88–3.64	1.79	0.75–4.30
Weekly working hours	≤35	1		1	
	>35	1.4	0.78–2.50	1.09	0.53–2.24
Unpaid overtime hours	none	1		1	
	<2 h per day	1.1	0.47–2.55	0.44	0.15–1.25
	≥2 h per day	**2.66**	**1.45–4.89**	**2.25**	**1.11–4.59**
Absence due to sickness during 2020	no	1		1	
	yes	**3.40**	**1.57–7.37**	**3.82**	**1.53–9.58**

cOR, crude odds ratio; CI, confidence interval; aOR, adjusted odds ratio. Reference category outcome = absence of ill health (GHQ-12 < 3). Bold: statistically significant at *p* > 0.05.

**Table 5:** Association between poor mental health and sociodemographic teacher-related variables and working conditions in Chilean teachers grouped by years of experience (n = 242). Chile, 2020 (E-TLD).

Characteristics	Categories	Years of Teaching Experience					
		>10 (n = 112)		6–10 (n = 84)		≤5 (n = 46)	
		aOR	CI (95%)	aOR	CI (95%)	aOR	CI (95%)
Gender	Male	1		1		1	
	Female	0.83	0.26–2.67	0.43	0.09–2.11	0.52	0.03–9.99
Age at entry into profession	>40	1		1		1	
	31–40	0.72	0.23–2.22	-	-	0.62	0.01–27.09
	≤30	-	-	**9.77**	**1.17–81.21**	-	-
Region	Bío-Bío	1		1		1	
	Ñuble	0.74	0.25–2.21	2.00	0.39–10.33	1.58	0.03–90.97
	Other	0.47	0.05–4.75	0.99	0.08–12.08	0.42	0.01–35.40
Type of school	Private	1		1		1	
	Private-subsidized	4.41	0.90–21.55	2.68	0.50–14.39	0.19	0.00–10.08
	Public	3.23	0.61–17.14	6.17	0.86–44.05	0.01	0.00–3.73
Educational teaching level	Secondary	1		1		1	
	Primary	0.71	0.27–1.89	2.02	0.51–8.02	0.3	0.03–3.04
	Pre-school	0.85	0.21–3.41	0.36	0.06–2.30	2.63	0.11–63.08
Head teacher	no	1		1		1	
	yes	0.58	0.23–1.46	0.56	0.12–2.62	1.76	0.14–22.25
Type of contract	Permanent	1		1		1	
	Fixed-term for >1 year	1.14	0.16–8.31	1.22	0.24–6.27	1.3	0.02–98.91
	Fixed-term for ≤1 year	1.29	0.24–6.81	1.37	0.25–7.71	**90.05**	**1.25–6473.93**
Weekly working hours	≤35	1		1		1	
	>35	2.09	0.64–6.79	**0.07**	**0.01–.51**	40.34	0.57–2831.63
Unpaid overtime hours	none	1		1		1	
	<2 h per day	0.53	0.08–3.62	0.74	0.08–7.02	0.15	0.00–34.84
	≥2 h per day	2.16	0.80–5.83	**6.11**	**1.09–34.30**	3.03	0.02–557.49
Absence due to sickness during 2020	no	1		1		1	
	yes	2.4	0.69–8.42	**6.72**	**1.04–43.61**	-	-

aOR, adjusted odds ratio; CI, confidence interval. Outcome reference category = absence of ill health (GHQ-12 < 3). Bold: statistically significant at *p* > 0.05.

**Table 6:** Association between poor mental health and sociodemographic teacher-related variables and working conditions in Chilean female’s teachers (n = 210). Chile, 2020 (E-TLD).

Characteristics	Categories	Female	
		aOR	CI (95%)
Age at entry into profession	>40	1	
	31–40	0.60	0.24–1.48
	≤30	-	-
Region	Bío-Bío	1	
	Ñuble	1.46	0.60–3.54
	Other	0.99	0.27–3.64
Type of school	Private	1	
	Private-subsidized	1.96	0.67–5.80
	Public	1.66	0.50–5.46
Educational teaching level	Secondary	1	
	Primary	0.70	0.33–1.50
	Pre-school	0.68	0.26–1.80
Head teacher	No	1	
	Yes	**0.48**	**0.23–1.01**
Years of teaching experience	>10	1	
	6–10	1.70	0.81–3.58
	≤5	1.85	0.73–4.66
Type of contract	Permanent	1	
	Fixed-term for >1 year	0.47	0.17–1.30
	Fixed-term for ≤1 year	2.56	0.93–7.06
Weekly working hours	≤35	1	
	>35	1.02	0.47–2.22
Unpaid overtime hours	none	1	
	<2 h per day	0.86	0.26–2.83
	≥2 h per day	**4.40**	**1.86–10.39**
Absence due to sickness during 2020	No	1	
	Yes	**3.08**	**1.19–7.97**

aOR, adjusted odds ratio; CI, confidence interval. Outcome reference category = absence of ill health (GHQ-12 < 3). Bold: statistically significant at *p* > 0.05.
